# Oxidative stress strongly restricts the effect of codon choice on the efficiency of protein synthesis in *Escherichia coli*

**DOI:** 10.3389/fmicb.2022.1042675

**Published:** 2022-11-29

**Authors:** Lorenzo Eugenio Leiva, Sara Elgamal, Sebastian A. Leidel, Omar Orellana, Michael Ibba, Assaf Katz

**Affiliations:** ^1^Programa de Biología Celular y Molecular, Instituto de Ciencias Biomédicas, Facultad de Medicina, Universidad de Chile, Santiago, Chile; ^2^Facultad de Ciencias, Universidad de Chile, Santiago, Chile; ^3^Schmid College of Science and Technology, Chapman University, Orange, CA, United States; ^4^Department of Microbiology, The Center for RNA Biology, Ohio State University, Columbus, OH, United States; ^5^Research Group for RNA Biochemistry, Department of Chemistry, Biochemistry and Pharmaceutical Sciences, University of Bern, Bern, Switzerland

**Keywords:** codon usage, oxidative stress, regulation of translation, *Escherichia coli*, protein synthesis

## Abstract

**Introduction:**

The response of enterobacteria to oxidative stress is usually considered to be regulated by transcription factors such as OxyR and SoxR. Nevertheless, several reports have shown that under oxidative stress the levels, modification and aminoacylation of tRNAs may be altered suggesting a role of codon bias in regulation of gene expression under this condition.

**Methods:**

In order to characterize the effects of oxidative stress on translation elongation we constructed a library of 61 plasmids, each coding for the green fluorescent protein (GFP) translationally fused to a different set of four identical codons.

**Results:**

Using these reporters, we observed that GFP production levels vary widely (~15 fold) when *Escherichia coli* K-12 is cultured in minimal media as a consequence of codon choice variations. When bacteria are cultured under oxidative stress caused by paraquat the levels of GFP produced by most clones is reduced and, in contrast to control conditions, the range of GFP levels is restricted to a ~2 fold range. Restricting elongation of particular sequences does not increase the range of GFP production under oxidative stress, but altering translation initiation rates leads to an increase in this range.

**Discussion:**

Altogether, our results suggest that under normal conditions the speed of translation elongation is in the range of the speed of initiation and, consequently, codon choice impacts the speed of protein synthesis. In contrast, under oxidative stress translation initiation becomes much slower than elongation, limiting the speed of translation such that codon choice has at most only subtle effects on the overall output of translation.

## Introduction

The medium and long term adaptation of bacteria to environmental changes requires an alteration of gene expression, which allows a modification of the abundance of each component of the proteome (Starosta et al., 2014; Browning and Busby, 2016; Zhu and Dai, 2020). Given its relevance for host-pathogen interactions (Slauch, 2011; Nguyen et al., 2017), several groups have studied such changes on gene expression under the effect of oxidative stress [reviewed at (Chiang and Schellhorn, 2012; Imlay, 2013, 2019; Zhu and Dai, 2020; Fasnacht and Polacek, 2021)]. It has been observed that in *Escherichia coli*, transcription is modulated by several key regulators, most importantly OxyR and SoxR (Zheng et al., 2001; Blanchard et al., 2007; Imlay, 2013). Furthermore, several alterations of translation have been found. Some of these are a result of the specific binding of small RNAs such as OxyS to or near to the ribosome binding site (RBS) of particular genes (Storz et al., 2004; Fröhlich and Gottesman, 2018). Global alterations to translation have also been observed. For example, we found that the increase in (p)ppGpp levels under oxidative stress suppresses Shine-Dalgarno (SD) led translation, while enhancing translation of genes with the initiation codon at or very near to the 5′ end of mRNA (leaderless mRNAs or lmRNAs; Leiva et al., 2021). In addition to regulation of translation initiation, elongation is also known to be altered under oxidative stress. For instance, an increased error rate of threonyl-tRNA synthetase has been observed to allow the enhanced incorporation of serine to the nascent peptide in sites coding for threonine in the mRNA (Ling and Söll, 2010). In contrast, the error rate of phenylalanyl-tRNA synthetase decreases, reducing the incorporation of m-tyrosine to phenylalanine sites (Steiner et al., 2019). Changes in the speed of codon translation have also been reported, although these alterations seem to be strain specific. In some strains such as BL21(DE3) and NCM3722 all tRNAs are degraded under oxidative stress, strongly decreasing the speed of translation elongation (Zhong et al., 2015; Zhu and Dai, 2020). In contrast, in strain MG1655 tRNAs are not degraded, but we have observed a specific inactivation of tRNA^Gly^ for the aminoacylation reaction, thereby altering the translation of Gly codons (Leiva et al., 2020).

Some of the aforementioned studies induce oxidative stress using 250 μM paraquat (Blanchard et al., 2007; Leiva et al., 2020, 2021), a cycling agent that oxidizes intracellular redox cofactors such as NADPH and partially reduces oxygen to superoxide radical (Hassan and Fridovich, 1979). Together, these studies have shown that when exposed to 250 μM paraquat, *E. coli* MG1655 changes its transcriptional program (Blanchard et al., 2007), inhibits SD-led translation while enhancing translation of lmRNA (Leiva et al., 2021) and alters translation of Gly codons (Leiva et al., 2020). Nevertheless, how the translation of the remaining codons is affected is currently unknown. In order to better understand how bacteria adapt to oxidative stress, we have studied in more detail the effects of oxidative stress on translation elongation. To that end, we have studied the expression of several fluorescent reporters enriched with diverse codons under non-stress, “normal,” conditions and oxidative stress caused by 250 μM paraquat. Unexpectedly, we have found that while codon choice is relevant for controlling translation efficiency under control conditions, it shows little relevance under oxidative stress in *E. coli* K-12 MG1655. This change seems to be a consequence of the inhibition of translation initiation that is more limiting for the translation process under stress than what is observed under control conditions.

## Materials and methods

### Strains and culture media

All experiments of this work were performed using *E. coli* K-12 MG 1655 or its ∆*efp*::FRT derivative. The Δ*efp*::*kan* mutation was transduced with P1vir from *E. coli* BW25113 *efp*::*kan* (Tollerson et al., 2018) to *E. coli* K-12 MG1655, and transformed with thermosensitive plasmid pCP20 to excise the kanamycin resistance gene (MG1655 Δ*efp*::FRT; Datsenko and Wanner, 2000). M9 minimal medium (47.7 mM Na_2_HPO_4_, 22.0 mM KH_2_PO_4_, 8.6 mM NaCl, 18.7 mM NH_4_Cl, 2 mM MgSO_4_, 0.1 mM CaCl_2_, and 0.4% Glycerol) was used for all cultures of *E. coli*. Unless otherwise indicated, M9 media was supplemented with branched amino acids (M9br; isoleucine, leucine, and valine 50 μg/ml each). When indicated, 0.4% arabinose, 100 μg/ml ampicillin or 250 μM paraquat were added to the culture media.

### Construction of GFP/mCherry reporters and prediction of secondary structure of the 5′ end

Reporter plasmids enriched in codons for Gly, Glu, and Ala have been previously constructed (Rojas et al., 2018; Leiva et al., 2020). Reporter plasmids enriched in codons coding for the remaining 17 amino acids as well as two control plasmids with four randomly selected non-identical codons were cloned using a similar procedure. Briefly, the parental plasmid, pBAD30SFIT contains a tandem fluorescent transcriptional fusion cassette composed of superfolder green fluorescent protein (sfGFP) followed by a modified mCherry, itag-mCherry. The plasmid contains a XhoI-SpeI site after the 3rd codon of *sfgfp* where tetra codon sequences were inserted using annealed oligonucleotide cloning with the oligonucleotide pairs described in Supplementary Table S1 (Leiva et al., 2020). Some of these reporters have been used in previous studies (Rojas et al., 2018; Leiva et al., 2020). Furthermore, annealed oligonucleotides enriched for the CTC and CTG codons (oligonucleotides 59 and 61 in Supplementary Table S1) were inserted into pSD and plmRNA plasmids (Leiva et al., 2021), two variants of pBAD30SFIT designed to generate a leader transcript (42-nucleotides containing an SD sequence) and a leaderless transcript, respectively. Secondary structure of segments of the 5′ end of the produced mRNAs (57, 100, and 140 nucleotides long) were predicted using NUPACK with the default parameters (Zadeh et al., 2011).

### Measurement of GFP and mCherry expression

GFP and mCherry fluorescence were determined using previously published protocols (Leiva et al., 2020) with minor modifications. Briefly, M9br media with ampicillin (100 μg/ml) was inoculated with bacteria from saturated overnight cultures in M9 media supplemented with 0.1% tryptone and incubated at 37°C with shaking. At an OD_600_ of 0.4–0.6 (mid-log phase) a 50 μl aliquot was mixed with 150 μl fresh M9 media supplemented with arabinose (0.4% final concentration) in a 96-well optical-bottom plate. When indicated, media additionally contained paraquat (250 μM final concentration). Plates were further shaken at 37°C in a microplate reader (INFINITE M200PRO, TECAN) where OD_600_ and fluorescence intensity of GFP (Ex. 480 ± 4.5 nm, Em. 515 ± 10 nm) and mCherry (Ex. 555 ± 4.5 nm, Em. 600 ± 10 nm) were determined. To determine the background levels of fluorescence, in each experiment a strain transformed with pBAD30 (parental plasmid of pS1, devoid of genes coding for GFP and mCherry) was analyzed in all tested conditions using the same protocol as with the experimental samples. The autofluorescence detected at different OD_600_ using that control strain was used to graph the background level according to OD_600_. The GFP background vs. OD_600_ fitted an exponential curve, while the mCherry background fitted a linear curve. The respective curve equations were used to estimate and subtract the GFP and mCherry background fluorescence at different OD_600_ for each sample during kinetics. Quantifications reported in this work were performed 2 h after induction. At this time point and using the reporter strain with the parental plasmid (S1), the GFP fluorescence signal was 20.1% ± 4.8% of the maximum (plateau) signal in control conditions and 34.2 ± 1.1 under stress conditions. The fluorescence signal of mCherry was 8.8% ± 2.0% of the maximum signal under control conditions and 34.5% ± 5.6% under oxidative stress conditions. The four values were within the initial linear segment of fluorescence appearance in time (Supplementary Figure S1).

## Results

### Measurement of the impact of codon choice on translation efficiency

Previously, we have shown that under oxidative stress caused by paraquat or hydrogen peroxide, tRNA^Gly^ is partially inactivated in *E. coli* K-12 MG1655, while other tRNAs remain as active as observed under control conditions. We further found that in addition to tRNA^Gly^ inactivation, paraquat stress induces a change in Gly codon preference. While under control conditions GGA was the least efficient Gly codon, under 250 μM paraquat this codon is translated at a similar efficiency as the other three codons for this amino acid (Leiva et al., 2020). Although tRNA^Gly^ was the only tRNA found to be inactivated under these conditions, codon translation efficiency may change due to several other factors. Most importantly, codon translation may be altered by changes in the fraction of a tRNA that is aminoacylated (e.g., due to changes in amino acid availability; Subramaniam et al., 2013a; Katz et al., 2016) or because of chemical modifications of the tRNA anticodon loop (Buck and Griffiths, 1982; Katz et al., 2016).

**Figure 1:**
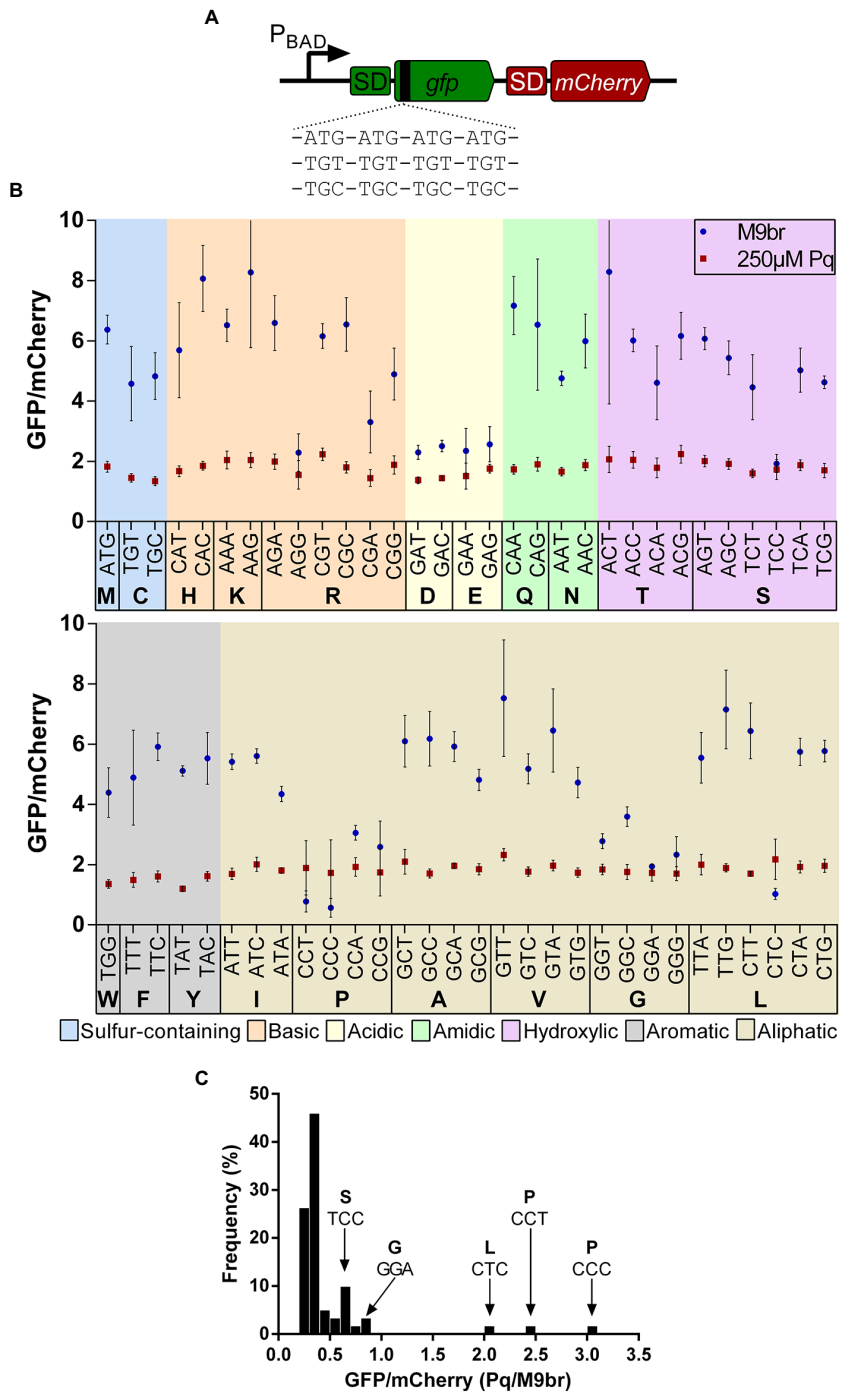
Effect of codons on the production of GFP in control and oxidative stress conditions. **(A)** A scheme of the reporter library used to test the effects of codons in translation under control and oxidative stress conditions. In each reporter 4 codon repetitions were inserted after the fourth codon of *gfp* that is cloned in transcriptional fusion upstream of the gene coding for mCherry. Each reporter plasmid was transformed into *E. coli* K-12 MG1655. Cells were cultured in minimal media supplemented with branched amino acids under control or oxidative stress conditions induced by 250 μM paraquat. In both conditions expression of GFP and mCherry was induced and fluorescence of both proteins was quantified after 2 h of induction. The graph in **(B)** shows the average fluorescence of GFP normalized by the fluorescence of mCherry for each reporter strain under control and oxidative stress conditions. **(C)** The ratio between the values observed in stress and control conditions were calculated and a histogram was constructed with the number of reporters presenting each ratio using bins of 0.1 units.

In order to determine whether the efficiency of translation of other codons is also altered under oxidative stress, we designed a library of 61 reporter plasmids. Each of these plasmids encode an operon composed of the genes coding for GFP followed by mCherry in transcriptional fusion. A different set of four contiguous identical codons is introduced between the fourth and fifth codons of GFP, so that each of the 61 reporters has a different amino acid coding codon added (Figure 1A). Changes on the efficiency of translation of such codons are expected to affect the synthesis of GFP if their speed of translation is similar or slower than that of translation initiation. In fact, our previous work has shown that addition of four codons is enough to report differences in the efficiency of translation of those codons (Elgamal et al., 2014; Rojas et al., 2018; Leiva et al., 2020). Also, we have observed that under these conditions the stability of different segments of the reporter mRNA is similar (Leiva et al., 2021). Therefore, we expect that changes in transcript level or alterations of translation initiation will affect the synthesis of GFP and mCherry in a similar manner. Thus, the changes in the ratio between GFP and mCherry fluorescence should mainly reflect the effects of alteration in GFP translation (Rojas et al., 2018; Leiva et al., 2020, 2021). In addition to the 61 reporters with four repeats of a single codon, two additional control reporters were constructed using four randomly selected codons. Finally, the parental plasmid [S1; (Elgamal et al., 2014; Rojas et al., 2018)] that contains two codons in the cloning site was used as an additional control.

### Efficiency of translation of 61 fluorescent reporters under control conditions

*E. coli* K-12 MG1665 transformed with each of the 61 reporter plasmids or each of the control plasmids were cultured in minimal media (M9) supplemented with glycerol and branched amino acids (M9br media) to prevent changes in translation in response to the known decreases in branched amino acids synthesis under oxidative stress (Imlay, 2008). At an OD_600_ of 0.6 two aliquots of the culture were taken. Expression of GFP and mCherry was induced in both aliquots by the addition of arabinose. However, while in the control culture the media continued to be M9br, in the “stress” aliquot, 250 μM paraquat was added to the media. All cultures were further incubated in 96 well plates, following OD_600_ as well as GFP and mCherry fluorescence. For all experiments, the fluorescence of GFP normalized by that of mCherry after 2 h of incubation was analyzed.

Under unstressed conditions, strains carrying the reporters enriched in glycine or proline codons were among the slowest to synthesize GFP, a result that is likely a consequence of the previously described lower speed of peptide bond formation using A site Pro or Gly charged tRNAs (Pavlov et al., 2009; Johansson et al., 2011; Chevance et al., 2014; Avcilar-Kucukgoze et al., 2016). In addition to Gly and Pro codons, we observed a slower production of GFP when the gene is enriched in acidic amino acids (Asp and Glu; Figure 1B; Supplementary Table S2). This results are in agreement with ribosome profiling experiments that show that Asp, IUPAC nomenclature of aminoacids. Pro and Gly codons are translated at a slower rate, although those experiments were performed in a richer medium containing all amino acids (Mohammad et al., 2019), a condition that likely affects tRNA aminoacylation levels (Avcilar-Kucukgoze et al., 2016).

Beyond the amino acids specific data, our analysis suggests that the efficiency of codon translation can strongly vary between codons that code for the same amino acid, although this is not observed for all amino acids. As proposed by Subramaniam et al., stronger variations between the efficiency of translation of GFP are observed for reporters enriched with amino acids encoded by a large number of codons (Subramaniam et al., 2013b). For example, the widest differences between the production of GFP by reporters enriched in codons coding the same amino acids are observed for Arg, Leu, and Ser. Each of these amino acids is coded by six codons. In average, we observed a 4.4-fold ratio between the most and the least efficient reporter enriched for each of these amino acids. In contrast, a similar analysis showed only an 1.2-fold average difference between the most and least efficient reporter in case of amino acids coded by two codons (Supplementary Figure S2). Some of the least efficient codons of these six codon groups have also been previously described as “slow” for *in vivo* translation [e.g., Arg AGG (Chevance et al., 2014)]. These differences in GFP production do not seem to be a consequence of altered 5′ UTR folding, as predictions of the secondary structure of this region do not reveal a clear alteration in the accessibility of the Shine-Dalgarno and initiation codon of the reporters that exhibit a lower GFP florescence (Supplementary Figure S3).

Finally, although in the case of some amino acids such as Val there is a relation between efficiency of GFP translation and frequency of codon usage under the non-stress condition, for most amino acids such as Leu or Ser we do not observe a clear correlation (Supplementary Figure S4), similar to what others have observed (Subramaniam et al., 2013b). Thus, as discussed in the previous paragraphs, the data obtained using our GFP/mCherry reporters confirm results that have been obtained by others using different strategies, thereby validating our approach.

### Effects of oxidative stress on codon translation

**Figure 2:**
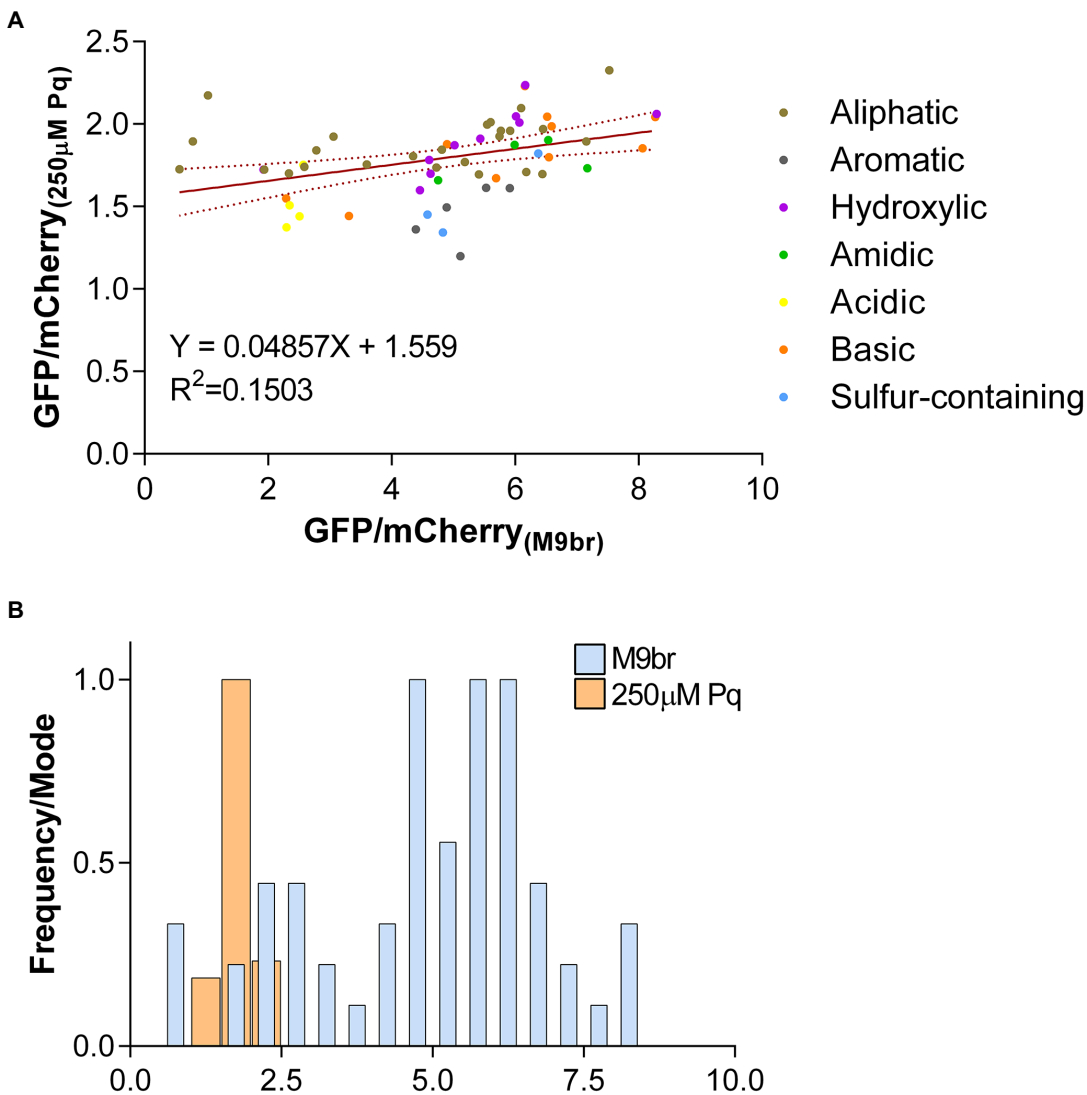
The effects of codon choice on the production of GFP are suppressed by oxidative stress. **(A)** The ratios between GFP and mCherry fluorescence for each reporter strain observed under oxidative stress conditions are plotted against the values observed under non-stress conditions. A linear tendency trend line is plotted in red with dotted lines representing the 95% confidence interval of the best-fit line **(B)** Histogram showing the number of reporters that present each GFP/mCherry fluorescence ratios under control and oxidative stress conditions. The total number of reporters in each range were normalized against the most frequent number for the condition (Frequency/Mode) so that the height of bars are similar for both conditions. Bins for both conditions are 0.5. Control bars are thinner to allow visualization of bars for the stress condition. Data presented in this figure is a different representation of data presented in Figure 1B and Supplementary Figure S4.

We next analyzed the effect of oxidative stress on the expression of *gfp* in our codon enrichment library. As expected from the known global inhibition of transcription and translation under oxidative stress, we observed that the levels of fluorescence generated in all reporter strains were much lower under oxidative stress than under non-stress conditions. For instance, the GFP and mCherry fluorescence for the strain carrying the parental S1 plasmid decreased ~92% and ~86%, respectively, after 2 h incubation in each of the tested media (Supplementary Table S3). Nevertheless, we observed different degrees of fluorescence reduction for each strain. For most reporters we observed a decrease in the ratio between GFP and mCherry fluorescence. Interestingly, for some we observed an increase or a maintenance of the fluorescence ratio under oxidative stress (Figures 1C, 2A). This behavior is observed mainly for codons that are inefficient under non-stress conditions such as CCC (Pro), CCT (Pro), CTC (Leu), GGA (Gly), and TCC (Ser). As a result of these changes, some of the least efficient codons under control conditions behave as “normal” codons under oxidative stress. Although some reporters show a smaller decrease in GFP/mCherry ratios than the S1 control (or even an increase), in most cases the ranking of efficiency of GFP production between the different codons that code for a single amino acid is maintained under oxidative stress. Hence, the most efficiently translated codon under control conditions, usually remains as the most efficiently translated codon under oxidative stress in the case of most amino acids (Supplementary Figure S4).

### Oxidative stress decreases the impact of elongation on GFP production

In addition to the evident decrease in expression of all reporters and the alteration in the efficiency ranking of reporters enriched on specific codons, oxidative stress strongly decreases the diversity of translation efficiencies that are observed under non-stress conditions. While under control conditions we observed a broad range of *gfp* translation efficiencies from about 0.57 to 8.3 times mCherry efficiency (~15 times between the least and most efficient reporters), under oxidative stress, the range is significantly reduced with GFP fluorescence values ranging between approximately 1.2 and 2.3 times mCherry fluorescence (~1.9 times between the least and most efficient reporters; Figure 2). Thus, while the change of only four adjacent codons may alter the efficiency of GFP synthesis by one order of magnitude under control conditions, its impact under oxidative stress is almost 8-fold smaller.

The fact that codon choice does not impact the synthesis of GFP under oxidative stress, strongly suggests that under this condition a different step becomes limiting for the *gfp* translation process. To confirm that elongation of translation has a smaller impact on protein synthesis under oxidative stress, we tested the effect of a strong inhibition of elongation on GFP production. To this end, we transformed the GFP reporters enriched with each of the 4 Pro codons as well as the parental plasmid (S1) in an *E. coli* strain lacking the gene coding for EF-P [*E. coli* K-12 MG1655 ∆*efp*::FRT (∆*efp*)]. EF-P is a translation elongation factor known to improve the translation of continuous proline codons that, in the absence of EF-P, induce translational pauses, which correspond to strong decreases of the speed of translation on particular mRNA locations (Doerfel et al., 2013; Ude et al., 2013; Elgamal et al., 2014; Hersch et al., 2014; Hummels and Kearns, 2020). In the wild type strain cultured under non-stress conditions, synthesis of GFP was similar to what we reported in Figures 1, 2, with a greater GFP production observed for the reporters enriched with commonly used Pro codons (CCA and CCG) than for reporters with rarely used Pro codons (CCT and CCC). Also in agreement with the results reported at Figures 1, 2, under oxidative stress the differences between reporters are lost, observing similar GFP/mCherry ratios for the four reporters enriched with Pro codons as well as for the parental plasmid. In contrast to these results, but in agreement with previous reports, we observed that in the ∆*efp* strain cultured under non-stress conditions, GFP production was much lower than mCherry production for the reporters containing 4 contiguous Pro codons. As a consequence, the ratio between GFP and mCherry fluorescence is much smaller for the reporters with 4 contiguous Pro codons than for the parental S1 plasmid. Nevertheless, under stress conditions, GFP production in the ∆*efp* strain is similar to that of mCherry and consequently, the GFP/mCherry ratios of the Pro enriched reporters is similar to what is observed for the parental plasmid. These results suggest that under oxidative stress there is a step of translation that is inhibited to a speed that is even slower than translation of poly-Pro in the absence of EF-P. Otherwise, poly-Pro reporters would be expected to have produced less GFP than the S1 reporter in the ∆*efp* strain under oxidative stress (Hersch et al., 2014).

### Changes to translation initiation have stronger impacts on GFP/mCherry production than changes to translation elongation

**Figure 3:**
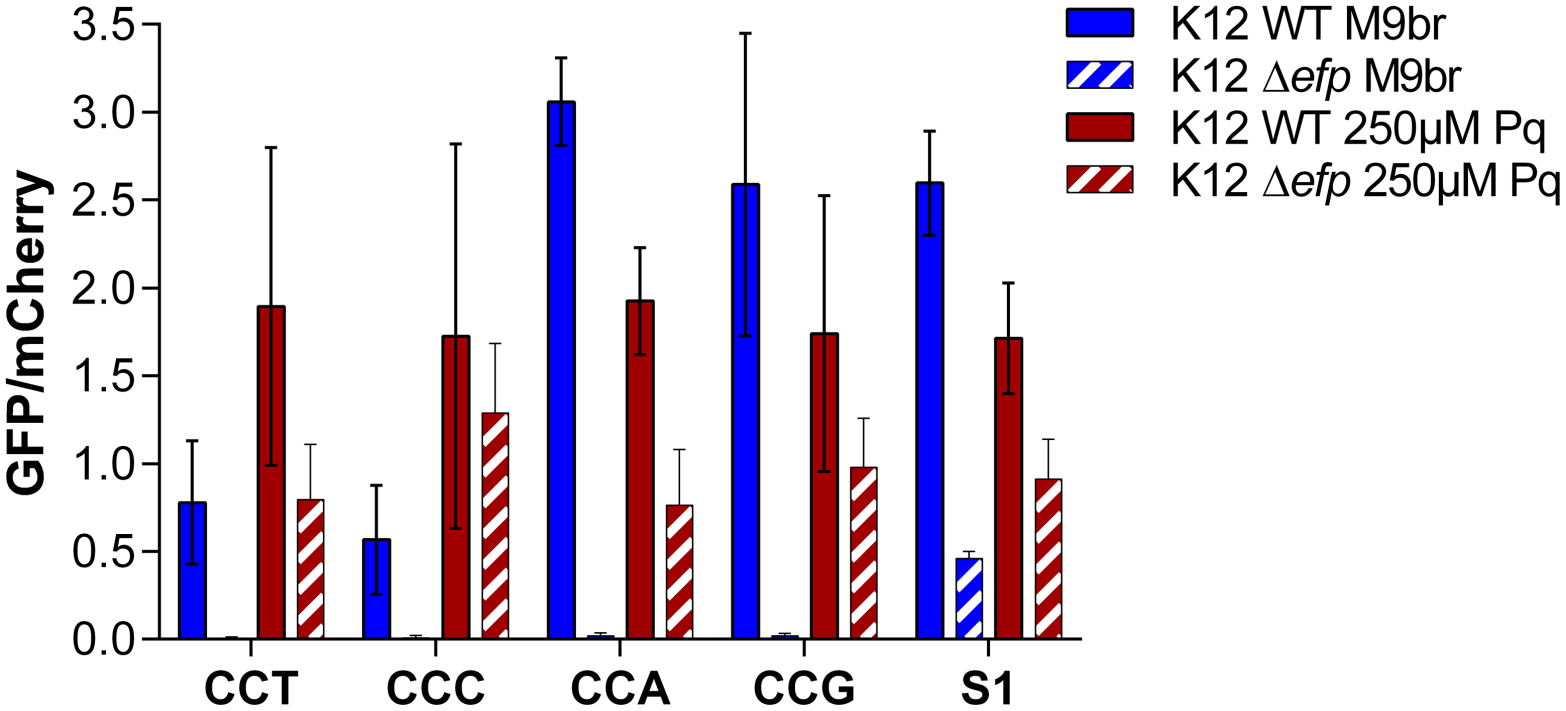
The strong inhibition of poly-proline codons translation caused by the deletion of *efp* does not affect translation under oxidative stress. Reporter plasmids enriched in proline codons were transformed in WT and Δ*efp E. coli* K-12 MG1655. GFP and mCherry fluorescence were subsequently measured under control and oxidative stress conditions. The differences in GFP production under control conditions are not observed under oxidative stress.

**Figure 4:**
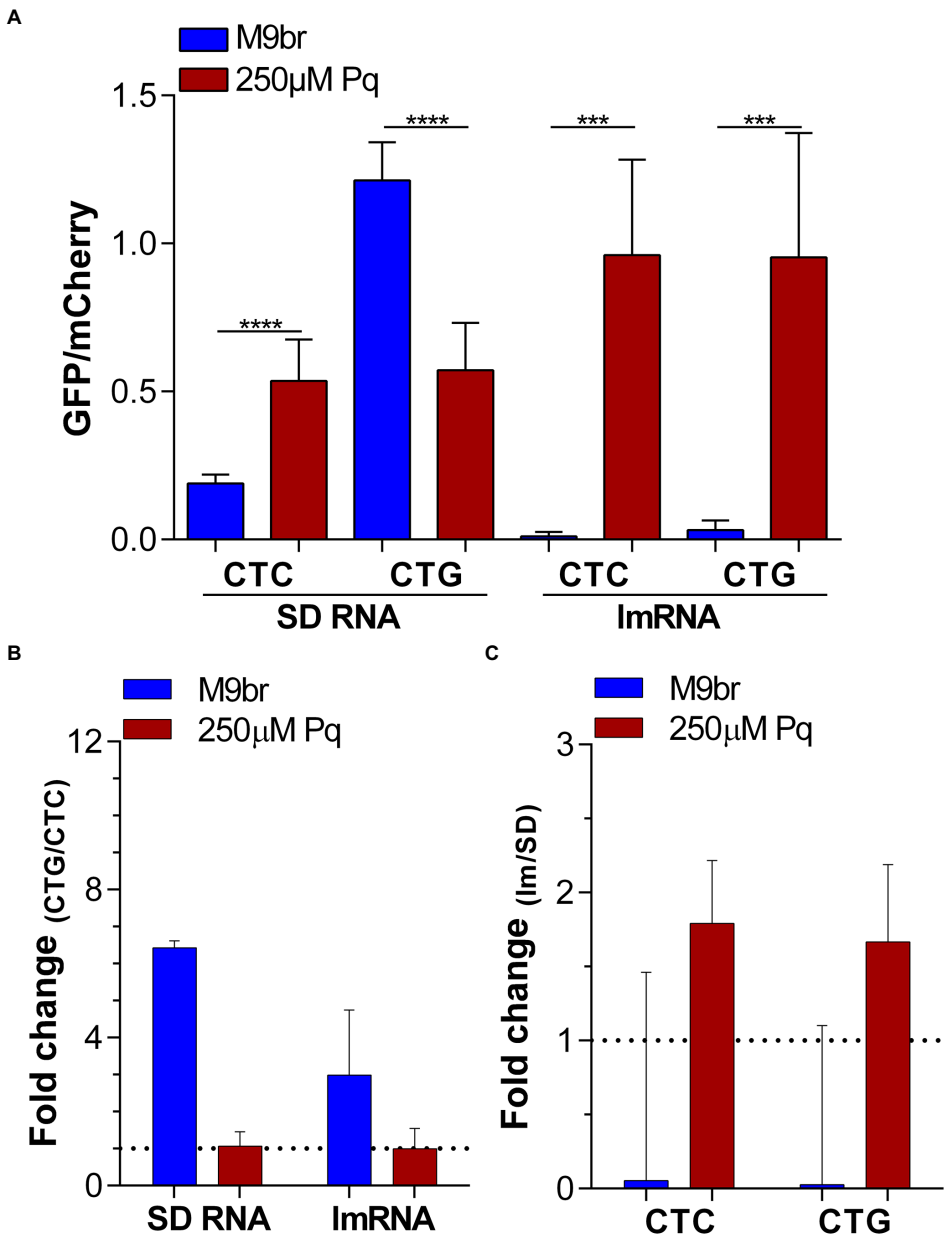
Altering translation initiation affects GFP synthesis under oxidative stress. **(A)** Ratios between GFP and mCherry synthesis under control and oxidative stress conditions were measured using reporters enriched in CTC and CTG Leu codons in the context of Shine-Dalgarno (SD RNA) and leaderless (lmRNA) translation. The ratios between these values are shown in **(B)** to highlight the effect of changing codons in the reporters and in **(C)** to highlight the effect of changing the translation initiation mechanism without altering codon choice. Statistical analyses: unpaired two tailed *t* test with Welch’s correction. ^****^p < 0.0001, ^***^p < 0.001.

As initiation is usually considered to be the slowest step of translation (Milón and Rodnina, 2012; Gualerzi and Pon, 2015), we hypothesized that under oxidative stress there is a much stronger decrease of initiation than of elongation of translation. If under control conditions the difference between the rate of translation initiation and elongation is small, then changes to elongation would have some impact on GFP synthesis (Hersch et al., 2014). In contrast, if under stress the rate of translation initiation suffers a stronger inhibition than elongation, then the impact of the latter on GFP synthesis would eventually be negligible. To test this hypothesis, we used 4 reporters that combine changes that affect the elongation step with changes that affect the initiation step of translation. To this end, we used the GFP/mCherry reporters that carry either four additional CTC or four additional CTG codons. These codons were selected because under non-stress conditions CTG allowed for a more efficient synthesis of GFP than CTC. However, under oxidative stress the efficiency of GFP synthesis by reporters enriched in these codons is very similar. In addition to the codon cluster, we altered the 5′ UTR of both reporters. In one version, named “SD,” the reporter uses a “canonical” 5′ UTR, including a Shine-Dalgarno sequence (SD) that allows for the efficient synthesis of GFP under control conditions. In the second or “lm” version, the 5′ UTR of both plasmids was mostly eliminated generating a leaderless mRNA (lmRNA). In all reporters the gene coding for mCherry uses a canonical SD led translation mechanism (Supplementary Figure S5). As *mCherry* translation initiated by the same mechanism in all reporters, we use it as an internal control for normalization. Although lmRNA translation of most genes is less efficient than SD translation under non-stress conditions, we have recently observed that under oxidative stress induced by 250 μM paraquat, lmRNA translation is activated (Leiva et al., 2021). Using these new set of SD reporters, we observed that changes in the selection of Leu codons added to the vector led to a 6.4-fold difference in GFP synthesis under non-stress conditions (Figure 3). Nevertheless, under oxidative stress, this difference strongly decreased to 1.1-fold (Figures 4A,B). Instead, changing initiation from the SD dependent to leaderless mechanism led to an increase of ~1.8-fold in GFP synthesis under oxidative stress (1.8-fold for CTC and 1.7-fold for CTG codon; Figures 4A,C). Thus, while codon choice do not have an effect on translation yield under oxidative stress indicating that is much faster than the limiting step, the choice of initiation mechanism do affect protein yield indicating that its speed is more limiting. Interestingly, under the non-stress condition, the effect of codon choice on the production of GFP was smaller when translation initiated by a leaderless-led mechanism than when using a SD-led mechanism (Figure 4B). This result is congruent with our interpretation. Previous reports have shown that the effect of pauses during the elongation step on translation yield is reduced as the speed of initiation decreases and turns more limiting to the process (Hersch et al., 2014). Thus, as leaderless initiation is slower than SD-led initiation under non-stress conditions (Leiva et al., 2021), it is expected that it will reduce the effect of faster steps of the process like elongation as observed in Figure 4B.

## Discussion

The bacterial response to hostile and stressful conditions requires an adaptation of the proteome to survive to such threats. This adaptation will depend on alterations to all the processes involved in defining protein concentrations, that is, transcription, translation and protein degradation. Under oxidative stress, bacterial cells suffer metabolic changes that are expected to indirectly affect many of these processes. For instance, ATP levels have been shown to decrease under at least some oxidative stress conditions (Barrette et al., 1989). This decrease is expected to impact initiation and elongation of both, translation and transcription. The increased concentration of (p)ppGpp will also affect both processes (Seither and Brown, 1984; Fitzsimmons et al., 2018). As previously mentioned, several steps of translation are in fact altered under oxidative stress by these or other reasons. Such alterations include global and specific changes to initiation as well as diverse changes to elongation efficiency and fidelity (Kojima et al., 2009; Ling and Söll, 2010; Liu et al., 2012; Wu et al., 2014; Nagano et al., 2015; Zhong et al., 2015; Yutthanasirikul et al., 2016; Willi et al., 2018; Steiner et al., 2019; Zhu and Dai, 2020). Nevertheless, under conditions where large changes are observed in all steps of gene expression, it is difficult to determine which of the changes will ultimately lead to a relevant impact on protein concentration, and thus, to bacterial adaptation. Our data strongly suggests that for *E. coli* K-12 MG1655 under oxidative stress caused by 250 μM paraquat, only changes to translation initiation will produce relevant alterations in protein levels, while changes to other steps of translation will only have minor effects. This does not mean that alterations of translation elongation are irrelevant, as any change to this step might still have an important impact on translation fidelity and protein folding, effectively altering the proportion of functional protein (Ling and Söll, 2010; Spencer et al., 2012; Hu et al., 2013; Bullwinkle et al., 2014; Wu et al., 2014; Buhr et al., 2016; Steiner et al., 2019; Liu et al., 2021). Nevertheless, while searching for mechanisms that regulate expression at the level of translation in *E. coli* K-12 MG1655, it is probable that researchers will identify mechanisms related to translation initiation rather than translation elongation. However, this might not be the case when studying other *E. coli* model strains such as *E. coli* BL21 or NCM3722. It has been proposed that in these strains the limiting step of translation under oxidative stress is elongation as a consequence of massive tRNA degradation (Zhong et al., 2015; Zhu and Dai, 2020). Thus, further research will be required to fully understand the strategies that bacteria like *E. coli* use to confront the hostile oxidative stress condition.

## Data availability statement

The original contributions presented in the study are included in the article/Supplementary material, further inquiries can be directed to the corresponding author.

## Author contributions

SE constructed the GFP/mCherry library and standardized initial conditions for its usage. LL performed most of the other experiments and data analyses. Data interpretation was performed by LL, OO, SL, MI, and AK. OO, SL, MI, and AK supervised experiments. AK wrote the paper with contributions from LL, SE, SL, OO, and MI. All authors contributed to the article and approved the submitted version.

## Funding

This research was funded by Fondo Nacional de Desarrollo Científico y Tecnológico, grant number 1191074 to AK and grant number 1190552 to OO; National Science Foundation, grant number MCB-2101998 to MI; Swiss National Science Foundation, grant number 310030_184947 to SL and a CONICYT Doctorado Nacional scholarship from Comisión Nacional de Investigación Científica y Tecnológica, grant number 21151441 to LL.

## Conflict of interest

The authors declare that the research was conducted in the absence of any commercial or financial relationships that could be construed as a potential conflict of interest.

## Publisher’s note

All claims expressed in this article are solely those of the authors and do not necessarily represent those of their affiliated organizations, or those of the publisher, the editors and the reviewers. Any product that may be evaluated in this article, or claim that may be made by its manufacturer, is not guaranteed or endorsed by the publisher.
