# Supplementary material for: Oxidative stress strongly restricts the effect of codon choice on the efficiency of protein synthesis in Escherichia coli
Source: Front Microbiol. 2022 Nov 29;13:1042675. doi: 10.3389/fmicb.2022.1042675 (PMC9749903; doi:10.3389/fmicb.2022.1042675)
Supplement: Supplementary file 1 [file Data_Sheet_1.PDF]

Supplementary information

Oxidative stress suppresses the effect of codon choice on the efficiency of protein synthesis

Lorenzo Eugenio Leiva[1,2], Sara Elgamal[4], Sebastian A. Leidel[5], Omar Orellana[1], Michael Ibba[3], Assaf Katz[1]*

[1] Programa de Biología Celular y Molecular, ICBM, Facultad de Medicina, Universidad de Chile, Santiago, Chile.

[2] Facultad de Ciencias, Universidad de Chile, Santiago, Chile

[3] Schmid College of Science and Technology, Chapman University, Orange, CA, United States

[4] Department of Microbiology and The Center for RNA Biology, Ohio State University, Columbus, Ohio, United States of America

[5] Research Group for RNA Biochemistry, Department of Chemistry, Biochemistry and Pharmaceutical Sciences, University of Bern, Bern, Switzerland

Current address:
Lorenzo Eugenio Leiva: Schmid College of Science and Technology, Chapman University, Orange, CA, United States
Sara Elgamal: Department of Internal Medicine, University of Cincinnati, Cincinnati, OH

Supplementary Tables

Table S1. Oligonucleotides used to construct codon enriched GFP reporters.

Clone	amino acid	codon	Forward oligonucleotide*	Reverse oligonucleotide*	Reference
1	Met	ATG	tcgag ATGATGATGATG a	ctagt CATCATCATCAT c	[This work]
2	Trp	TGG	tcgag TGGTGGTGGTGG a	ctagt CCACCACCACCA c	[This work]
3	Phe	TTT	tcgag TTTTTTTTTTTT a	ctagt AAAAAAAAAAAA c	[This work]
4	Phe	TTC	tcgag TTCTTCTTCTTC a	ctagt GAAGAAGAAGAA c	[This work]
5	Tyr	TAT	tcgag TATTATTATTAT a	ctagt ATAATAATAATA c	[This work]
6	Tyr	TAC	tcgag TACTACTACTAC a	ctagt GTAGTAGTAGTA c	[This work]
7	Cys	TGT	tcgag TGTTGTTGTTGT a	ctagt ACAACAACAACA c	[This work]
8	Cys	TGC	tcgag TGCTGCTGCTGC a	ctagt GCAGCAGCAGCA c	[This work]
9	His	CAT	tcgag CATCATCATCAT a	ctagt ATGATGATGATG c	[This work]
10	His	CAC	tcgag CACCACCACCAC a	ctagt GTGGTGGTGGTG c	[This work]
11	Gln	CAA	tcgag CAACAACAACAA a	ctagt TTGTTGTTGTTG c	[This work]
12	Gln	CAG	tcgag CAGCAGCAGCAG a	ctagt CTGCTGCTGCTG c	[This work]
13	Asn	AAT	tcgag AATAATAATAAT a	ctagt ATTATTATTATT c	[This work]
14	Asn	AAC	tcgag AACAACAACAAC a	ctagt GTTGTTGTTGTT c	[This work]
15	Lys	AAA	tcgag AAAAAAAAAAAA a	ctagt TTTTTTTTTTTT c	[This work]
16	Lys	AAG	tcgag AAGAAGAAGAAG a	ctagt CTTCTTCTTCTT c	[This work]
17	Asp	GAT	tcgag GATGATGATGAT a	ctagt ATCATCATCATC c	[This work]
18	Asp	GAC	tcgag GACGACGACGAC a	ctagt GTCGTCGTCGTC c	[This work]
19	Glu	GAA	tcgag GAAGAAGAAGAA a	ctagt TTCTTCTTCTTC c	[Rojas *et al*., 2018]
20	Glu	GAG	tcgag GAGGAGGAGGAG a	ctagt CTCCTCCTCCTC c	[Rojas *et al*., 2018]
21	Ile	ATT	tcgag ATTATTATTATT a	ctagt AATAATAATAAT c	[This work]
22	Ile	ATC	tcgag ATCATCATCATC a	ctagt GATGATGATGAT c	[This work]
23	Ile	ATA	tcgag ATAATAATAATA a	ctagt TATTATTATTAT c	[This work]
24	Pro	CCT	tcgag CCTCCTCCTCCT a	ctagt AGGAGGAGGAGG c	[This work]
25	Pro	CCC	tcgag CCCCCCCCCCCC a	ctagt GGGGGGGGGGGG c	[This work]
26	Pro	CCA	tcgag CCACCACCACCA a	ctagt TGGTGGTGGTGG c	[This work]
27	Pro	CCG	tcgag CCGCCGCCGCCG a	ctagt CGGCGGCGGCGG c	[This work]
28	Thr	ACT	tcgag ACTACTACTACT a	ctagt AGTAGTAGTAGT c	[This work]
29	Thr	ACC	tcgag ACCACCACCACC a	ctagt GGTGGTGGTGGT c	[This work]
30	Thr	ACA	tcgag ACAACAACAACA a	ctagt TGTTGTTGTTGT c	[This work]
31	Thr	ACG	tcgag ACGACGACGACG a	ctagt CGTCGTCGTCGT c	[This work]
32	Ala	GCT	tcgag GCTGCTGCTGCT a	ctagt AGCAGCAGCAGC c	[Rojas *et al*., 2018]
33	Ala	GCC	tcgag GCCGCCGCCGCC a	ctagt GGCGGCGGCGGC c	[Rojas *et al*., 2018]
34	Ala	GCA	tcgag GCAGCAGCAGCA a	ctagt TGCTGCTGCTGC c	[Rojas *et al*., 2018]
35	Ala	GCG	tcgag GCGGCGGCGGCG a	ctagt CGCCGCCGCCGC c	[Rojas *et al*., 2018]
36	Val	GTT	tcgag GTTGTTGTTGTT a	ctagt AACAACAACAAC c	[This work]

37	Val	GTC	tcgag GTCGTCGTCGTC a	ctagt GACGACGACGAC c	[This work]
38	Val	GTA	tcgag GTAGTAGTAGTA a	ctagt TACTACTACTAC c	[This work]
39	Val	GTG	tcgag GTGGTGGTGGTG a	ctagt CACCACCACCAC c	[This work]
40	Gly	GGT	tcgag GGTGGTGGTGGT a	ctagt ACCACCACCACC c	[Leiva *et al*. 2020]
41	Gly	GGC	tcgag GGCGGCGGCGGC a	ctagt GCCGCCGCCGCC c	[Leiva *et al*. 2020]
42	Gly	GGA	tcgag GGAGGAGGAGGA a	ctagt TCCTCCTCCTCC c	[Leiva *et al*. 2020]
43	Gly	GGG	tcgag GGGGGGGGGGGG a	ctagt CCCCCCCCCCCC c	[Leiva *et al*. 2020]
44	Ser	AGT	tcgag AGTAGTAGTAGT a	ctagt ACTACTACTACT c	[This work]
45	Ser	AGC	tcgag AGCAGCAGCAGC a	ctagt GCTGCTGCTGCT c	[This work]
46	Ser	TCT	tcgag TCTTCTTCTTCT a	ctagt AGAAGAAGAAGA c	[This work]
47	Ser	TCC	tcgag TCCTCCTCCTCC a	ctagt GGAGGAGGAGGA c	[This work]
48	Ser	TCA	tcgag TCATCATCATCA a	ctagt TGATGATGATGA c	[This work]
49	Ser	TCG	tcgag TCGTCGTCGTCG a	ctagt CGACGACGACGA c	[This work]
50	Arg	AGA	tcgag AGAAGAAGAAGA a	ctagt TCTTCTTCTTCT c	[This work]
51	Arg	AGG	tcgag AGGAGGAGGAGG a	ctagt CCTCCTCCTCCT c	[This work]
52	Arg	CGT	tcgag CGTCGTCGTCGT a	ctagt ACGACGACGACG c	[This work]
53	Arg	CGC	tcgag CGCCGCCGCCGC a	ctagt GCGGCGGCGGCG c	[This work]
54	Arg	CGA	tcgag CGACGACGACGA a	ctagt TCGTCGTCGTCG c	[This work]
55	Arg	CGG	tcgag CGGCGGCGGCGG a	ctagt CCGCCGCCGCCG c	[This work]
56	Leu	TTA	tcgag TTATTATTATTA a	ctagt TAATAATAATAA c	[This work]
57	Leu	TTG	tcgag TTGTTGTTGTTG a	ctagt CAACAACAACAA c	[This work]
58	Leu	CTT	tcgag CTTCTTCTTCTT a	ctagt AAGAAGAAGAAG c	[This work]
59	Leu	CTC	tcgag CTCCTCCTCCTC a	ctagt GAGGAGGAGGAG c	[This work]
60	Leu	CTA	tcgag CTACTACTACTA a	ctagt TAGTAGTAGTAG c	[This work]
61	Leu	CTG	tcgag CTGCTGCTGCTG a	ctagt CAGCAGCAGCAG c	[This work]
C1	Asn-Tyr-His-Gln	AAC TAC CAC CAG	tcgag AACTACCACCAG a	ctagt CTGGTGGTAGTT c	[This work]
C2	Trp-Glu-Phe-Asn	TGG GAA TTC AAT	tcgag TGGGAATTCAAT a	ctagt ATTGAATTCCCA c	[This work]

*XhoI and SpeI sites are marked in lower case

Supplementary table S2. GFP/mCherry ratio for each reporter strain cultured at M9br or M9br plus 250 μM paraquat.

Side chain	Amino acid	Codon	Frequency of usage*	M9br		M9br + Pq 250 μM	
				Average	Standard deviation	Average	Standard deviation
Sulfur containing	Met	ATG	0.0197	6.375	0.480	1.821	0.180
	Cys	TGT	0.0010	4.578	1.236	1.450	0.141
		TGC	0.0030	4.829	0.774	1.342	0.146
Basic	His	CAT	0.0044	5.691	1.579	1.671	0.177
		CAC	0.0138	8.066	1.096	1.853	0.148
	Lys	AAA	0.0600	6.518	0.537	2.044	0.295
		AAG	0.0191	8.272	2.496	2.042	0.247
	Arg	AGA	0.0001	6.592	0.910	1.910	0.120
		AGG	0.0000	2.292	0.624	1.550	0.475
		CGT	0.0488	6.157	0.411	2.230	0.208
		CGC	0.0187	6.548	0.891	1.799	0.190
		CGA	0.0003	3.308	1.028	1.443	0.278
		CGG	0.0001	4.896	0.858	1.879	0.300
Acid	Asp	GAT	0.0185	2.298	0.230	1.374	0.112
		GAC	0.0385	2.507	0.192	1.441	0.074
	Glu	GAA	0.0542	2.348	0.739	1.507	0.430
		GAG	0.0155	2.568	0.586	1.754	0.142
Amide	Gln	CAA	0.0046	7.169	0.966	1.733	0.156
		CAG	0.0304	6.536	2.178	1.903	0.226
	Asn	AAT	0.0032	4.755	0.236	1.659	0.143
		AAC	0.0371	5.993	0.894	1.873	0.183
Hydroxyl	Thr	ACT	0.0253	8.292	4.386	2.062	0.437
		ACC	0.0269	6.014	0.371	2.047	0.276
		ACA	0.0010	4.606	1.229	1.782	0.325
		ACG	0.0034	6.166	0.777	2.237	0.296
	Ser	AGT	0.0012	6.071	0.363	2.009	0.192
		AGC	0.0091	5.436	0.558	1.912	0.181
		TCT	0.0169	4.456	1.081	1.599	0.142
		TCC	0.0126	1.926	0.304	1.724	0.331
		TCA	0.0009	5.022	0.731	1.872	0.181
		TCG	0.0005	4.628	0.208	1.698	0.235
Aromatic	Trp	TGG	0.0061	4.393	0.822	1.362	0.141
	Phe	TTT	0.0069	4.889	1.578	1.494	0.245
		TTC	0.0260	5.913	0.457	1.611	0.183
	Tyr	TAT	0.0055	5.114	0.169	1.199	0.097
		TAC	0.0192	5.528	0.863	1.614	0.162
Neutral	Ile	ATT	0.0108	5.415	0.257	1.695	0.184
		ATC	0.0475	5.673	0.121	2.011	0.240
		ATA	0.0000	4.347	0.250	1.835	0.043
	Pro	CCT	0.0038	0.778	0.352	1.894	0.904
		CCC	0.0003	0.566	0.311	1.725	1.095
		CCA	0.0038	3.059	0.249	1.925	0.306
		CCG	0.0264	2.588	0.860	1.741	0.785
	Ala	GCT	0.0470	6.097	0.855	2.097	0.411
		GCC	0.0080	6.178	0.906	1.709	0.152
		GCA	0.0272	5.919	0.497	1.960	0.096
		GCG	0.0201	4.816	0.353	1.846	0.186
	Val	GTT	0.0499	7.527	1.938	2.326	0.205
		GTC	0.0063	5.186	0.499	1.770	0.156
		GTA	0.0227	6.455	1.378	1.969	0.179
		GTG	0.0120	4.727	0.506	1.736	0.153
	Gly	GGT	0.0536	2.781	0.251	1.841	0.174
		GGC	0.0354	3.598	0.324	1.679	0.119
		GGA	0.0005	1.937	0.087	1.724	0.270
		GGG	0.0015	2.334	0.598	1.700	0.235
	Leu	TTA	0.0005	5.546	0.839	1.997	0.339
		TTG	0.0026	7.152	1.305	1.895	0.141
		CTT	0.0029	6.440	0.925	1.696	0.077
		CTC	0.0023	0.967	0.096	1.984	0.384
		CTA	0.0003	5.745	0.452	1.926	0.198
		CTG	0.0611	5.772	0.357	1.960	0.220

* Frequencies of codon usage in highly expressed genes, as reported by Thanaraj and Argos, 1996.

Supplementary table S3. Effect of 250 µM paraquat on GFP and mCherry expression from the S1 reporter.

	GFP fluorescence				mCherry fluorescence			
	M9br		M9br 250µM Pq		M9br		M9br 250µM Pq	
	Mean	SD	Mean	SD	Mean	SD	Mean	SD
Fluorescence at 2hr post induction	3198	692	245	53	1283	157	181	22
% inhibition at 2hr post induction (Fluorescence)	92.3%				85.9%			

Fluorescence/OD_{600} at 2hrs post induction	20521	911	3015	294	8437	1463	2323	552
% inhibition at 2hr post induction (Fluorescence/OD_{600})	85.3%				72.5%			

Supplementary Figures

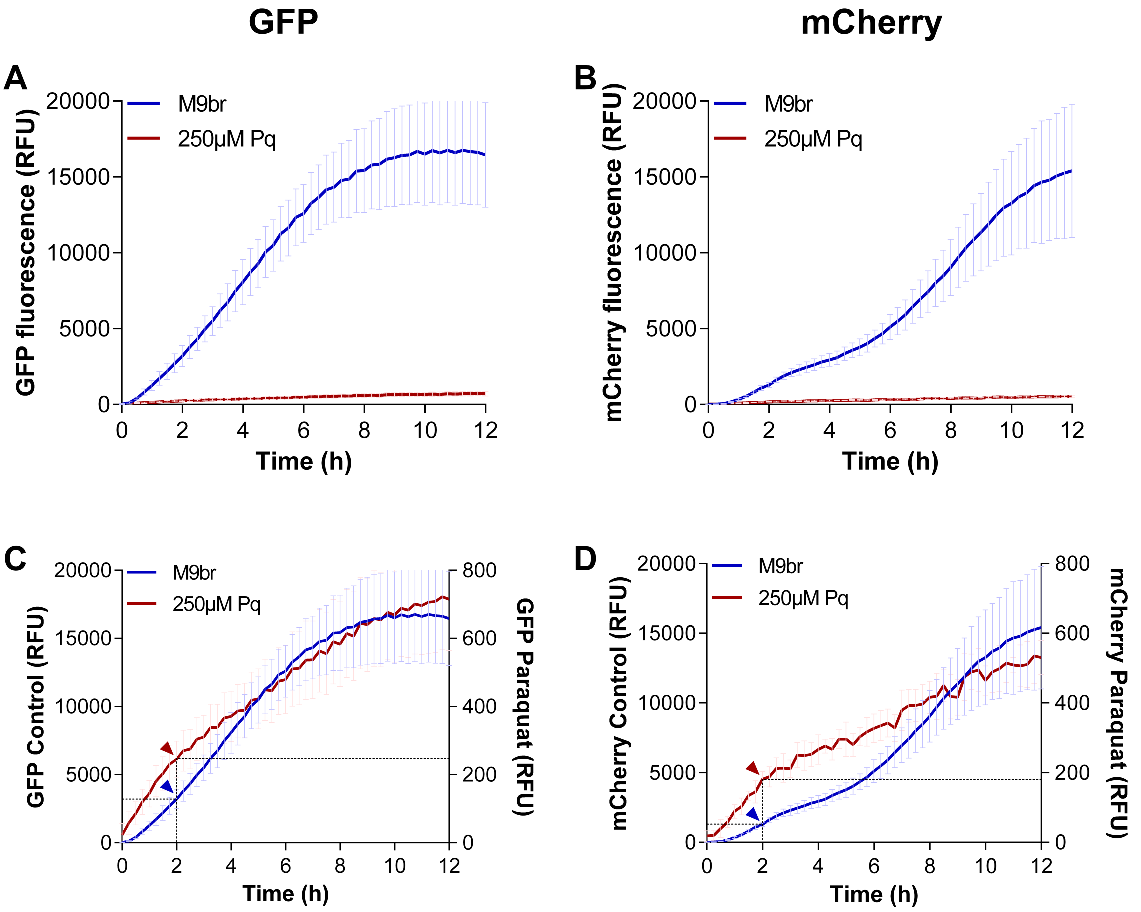

Figure S1. Kinetics of GFP and mCherry fluorescence production using the parental reporter strain cultured in M9br or M9br plus paraquat. *E. coli* K-12 MG1655 carrying the parental reporter plasmid (S1) was cultured in M9br media as indicated in the methods section. Following the addition of arabinose (blue lines) or arabinose plus 250 µM paraquat (red lines) GFP (**A and C**) and mCherry (**B and D**) fluorescence were measured. **A)** and **B)** show all data using the same scale, while **C)** and **D)** show the same data using a different scale for stress and no stress conditions. Data shown in all tables and figures correspond to the fluorescence measured 2 hrs after induction with arabinose. This time point is highlighted in **C)** and **D)** by blue (control) and a red (oxidative stress) triangles.

A)

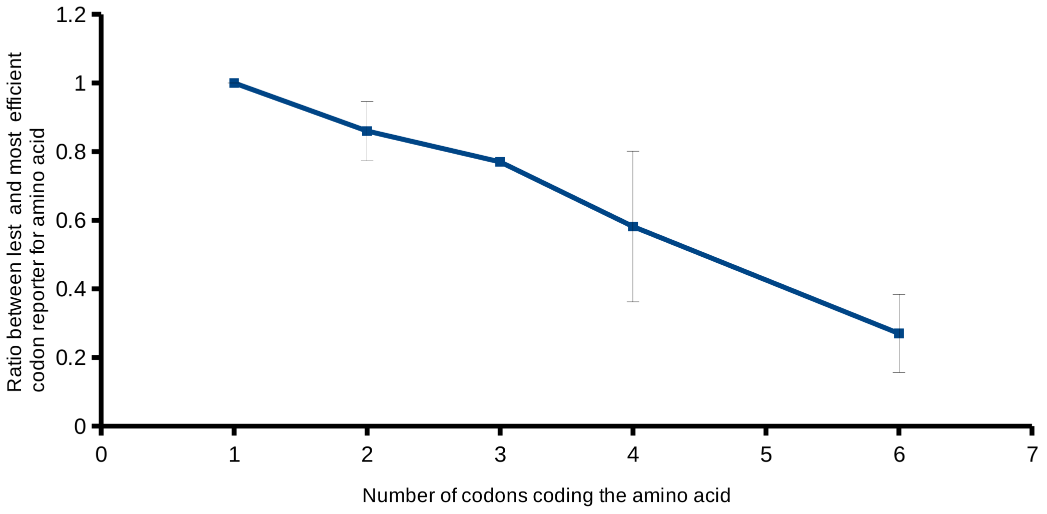

B)

N.° of codon	1			2			3			4			5			6		
	**aa**	**-/+**	**+/-**	**aa**	**-/+**	**+/-**	**aa**	**-/+**	**+/-**	**aa**	**-/+**	**+/-**	**aa**	**-/+**	**+/-**	**aa**	**-/+**	**+/-**
	Met	1	1	Cys	0.95	1.053	Ile	0.77	1.299	Ala	0.78	1.282	-	-	-	Arg	0.35	2.857
	Trp	1	1	Gln	0.91	1.099				Asn	0.79	1.266				Leu	0.14	7.143
				Glu	0.91	1.099				Gly	0.54	1.852				Ser	0.32	3.125
				His	0.71	1.408				Pro	0.19	5.263						
				Lys	0.79	1.266				Thr	0.56	1.786						
				Phe	0.83	1.205				Val	0.63	1.587						
				Tyr	0.92	1.087												

Figure S2. Ratio between the GFP/mCherry values of the least and most efficient reporters for each codon. GFP/mCherry ratios observed for reporters for each codon coding for the same amino acid as shown in figure 1B and table S2 were compared by dividing the smaller value by the highest value corresponding to each amino acid. **A)** Average value observed for all amino acids encoded by the same number of codons. Please note that for amino acids encoded by a single codon, we considered the ratio as 1. Also, as there is only one amino acid encoded by 3 codons, no standard deviation is shown for the corresponding point of the graph. **B)** Ratios observed for each amino acid. Columns “-/+” correspond to the ratios between the least and the most highly expressed GFP for reporters corresponding to each amino acid. Columns “+/-” correspond to the inverse value, that is, the ratios between the most and the least expressed GFP for reporters for each amino acid.

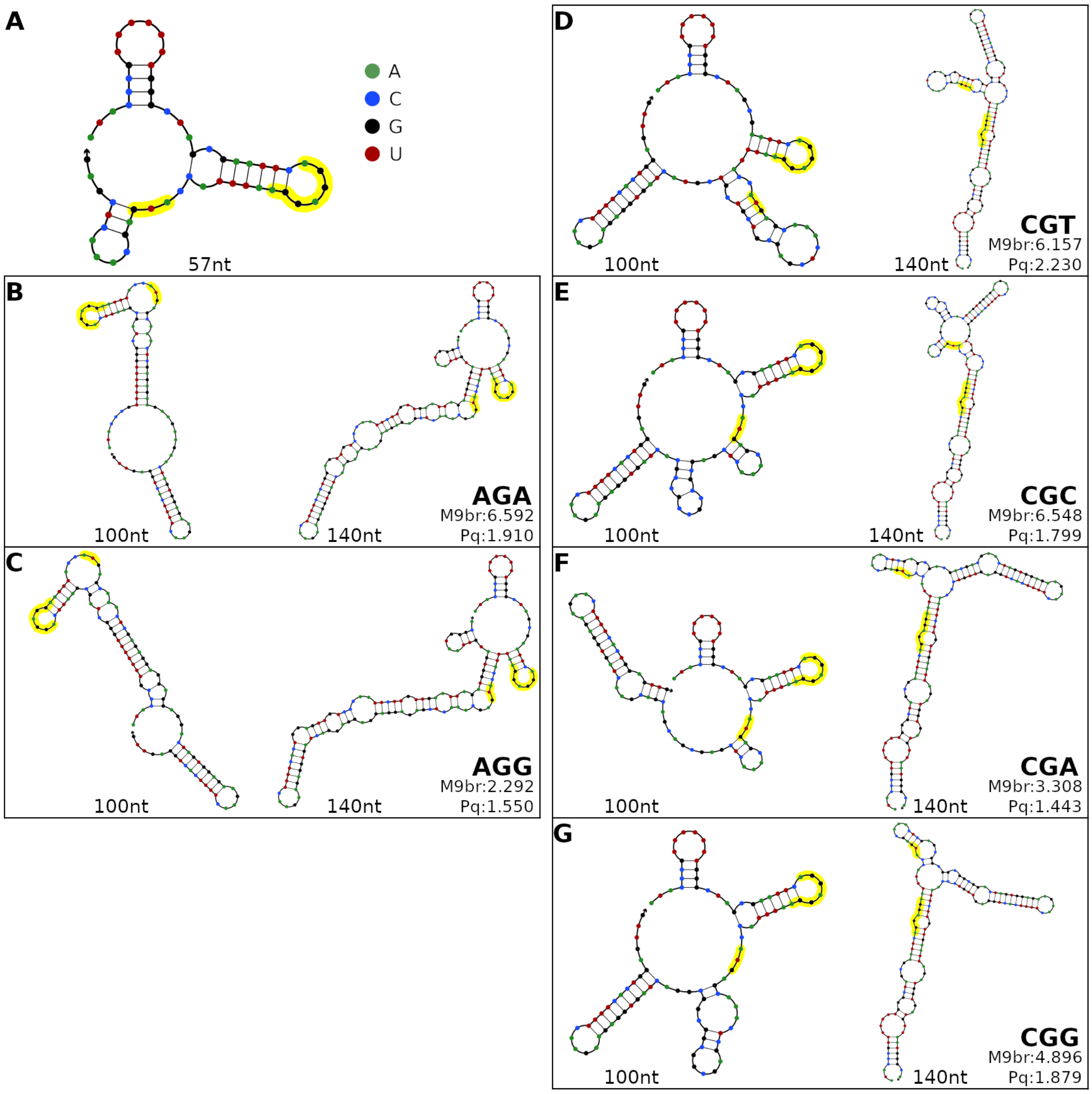

Figure S3. No correlation is observed between changes in GFP synthesis and alterations in the predicted structure of the 5' UTR as a consequence of codon changes. Structure of the 5' end of the reporter mRNAs enriched in Arg codons as predicted using the NUPACK software suite (Zadeh *et al*., 2011). **A)** Structure predicted for the first 57 nucleotides corresponding to the 5' UTR and the first 4 codons of *gfp*. As this fragment is common to all reporters of the library, a single structure is shown for all codons **B)** to **G)** shows the predicted structures of the first 100 (left) and 140 nucleotides (right) for each of the reporter variants enriched with Arg codons including the common segment, 12 nucleotides coding 4 copies of the corresponding codons and additional 31 or 71 nucleotides. The position of the Shine-Dalgarno (SD) sequence and of the initiation codon are highlighted in yellow. At the lower right segment of each panel, the codon identity and the GFP/mCherry fluorescence ratios at control (M9br) and oxidative stress (Pq) conditions are indicated. Note that RNAs where SD and initiation codon have similar accessibilities such as those enriched in AGA and AGG can have different GFP/mCherry fluorescence ratios. In contrast, other RNAs with dissimilar accessibility of the ribosomal binding site (RBS) such as AGA and CGC can have similar GFP/mCherry fluorescence ratios. Thus, differences in GFP/mCherry ratios do not appear to depend on changes to the 5' UTR structure.

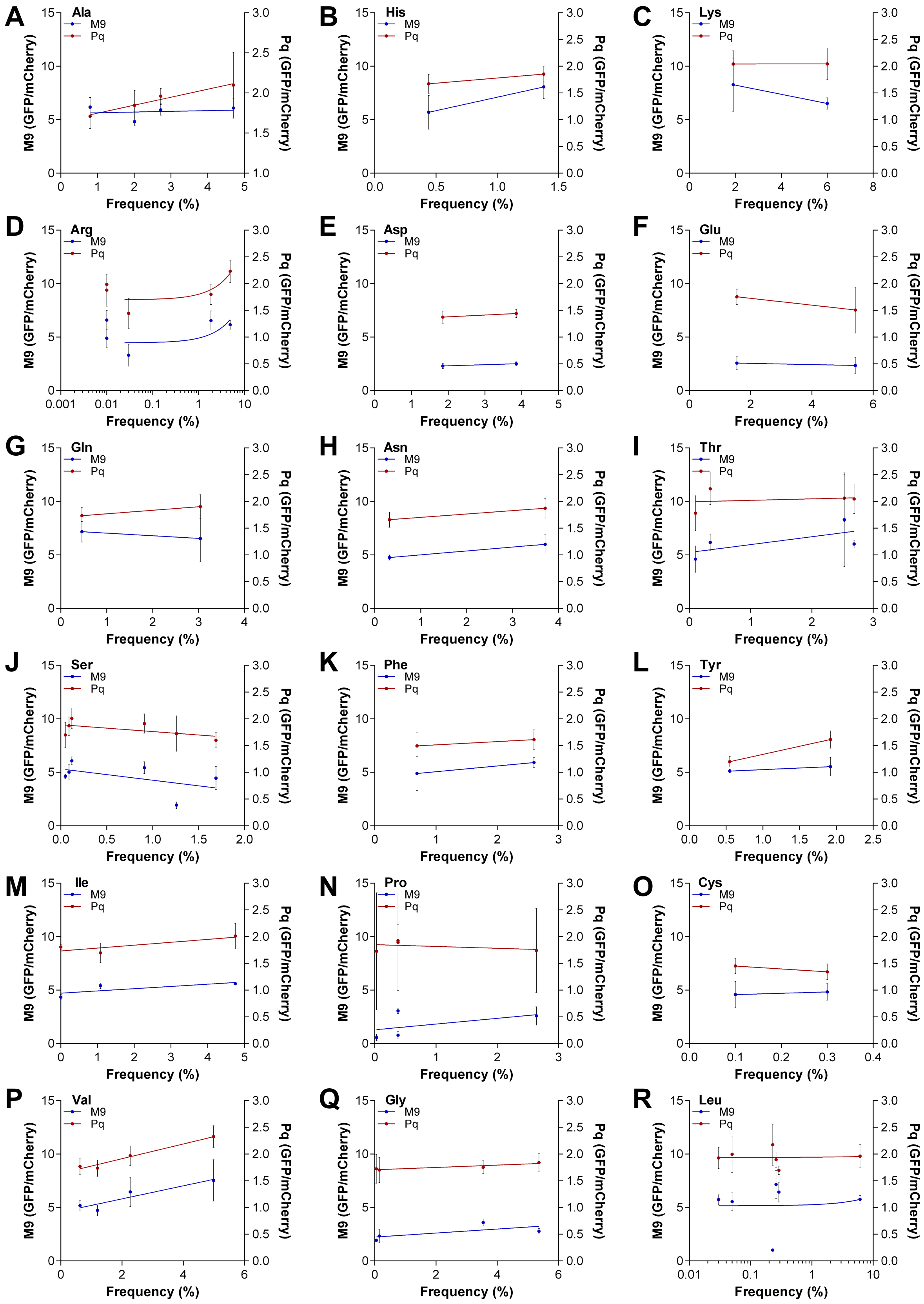

Figure S4. A correlation between the production of GFP reporters and the frequency of codon usage in highly expressed genes is observed only for a limited number of codons under control conditions. Comparison between the GFP/mCherry ratios observed for each reporter and the frequency of usage of the corresponding enriched codons as reported by

Thanaraj and Argos, 1996 is shown for each amino acid **A)** Alanine; **B)** Histidine; **C)** Lysine; **D)** Arginine; **E)** Aspartic acid; **F)** Glutamic acid; **G)** Glutamine; **H)** Asparagine; **I)** Threonine; **J)** Serine; **K)** Phenylalanine; **L)** Tyrosine; **M)** Isoleucine; **N)** Proline; **O)** Cysteine; **P)** Valine; **Q)** Glycine; **R)** Leucine. Data in this figure is a different representation of the data displayed in Figure 1B. Note that data obtained under stress and non-stress conditions is represented in different scales to allow an easier comparison.

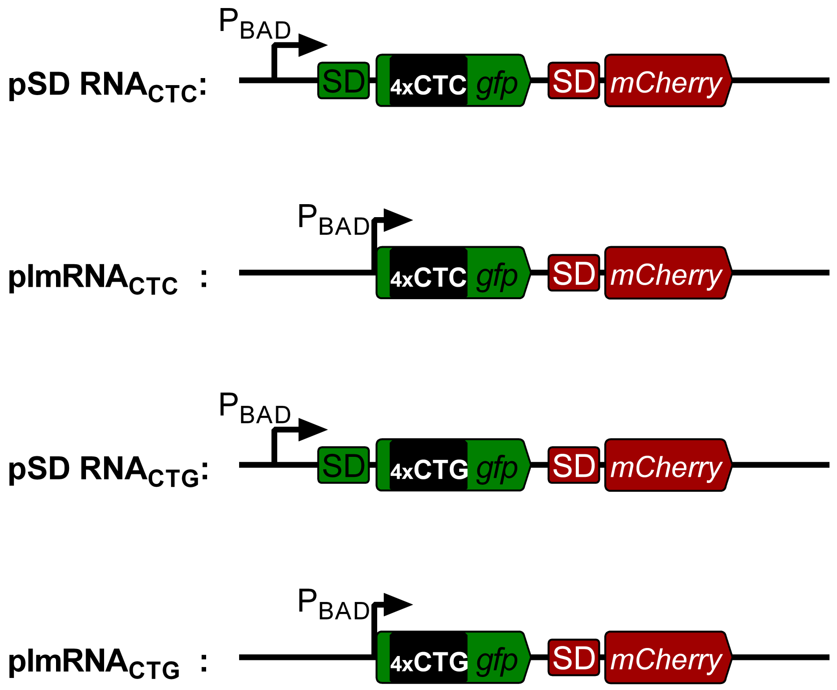

Figure S5. Schematic representation of SD- and leaderles-led translation reporters. Experiments presented in figure 4 were performed using four reporter plasmids carrying a bicistronic operon with the genes coding for GFP and mCherry in transcriptional fusion. The operons were under the control of the P_{BAD} promoter. In both pSD plasmids *gfp* translation was initiated using a SD-led mechanism, while a leaderless-led mechanism was used to initiate its translation in both lm reporters. Additionally, four continuous CTC codons were introduced between the XhoI and SpeI restriction sites in *gfp* of the "CTC" reporters, while four contiguous CTG codons were introduced in the same sites of the "CTG" reporters. *mCherry* translation initiated by SD-led translation in all reporters.
